# Supplementary material for: Polyketide synthase gene clusters in the ericoid mycorrhizal fungus Oidiodendron maius: genome-wide prediction, expression profiling, and functional characterization of knockout mutants
Source: Front Plant Sci. 2026 Sep 16;17:1924430. doi: 10.3389/fpls.2026.1924430 (PMC13623903; doi:10.3389/fpls.2026.1924430)
Supplement: Supplementary file 1 [file SupplementaryFile1.zip › Supplementary Materials/Supplementary Folder 1.zip!/Oidma1_AssemblyScaffolds_Repeatmasked/knownclusterblast/cluster13/jgi.p_Oidma1_39625_mibig_hits.html]

| MIBiG Protein | Description | MIBiG Cluster | MiBiG Product | % ID | % Coverage | BLAST Score | E-value |
| --- | --- | --- | --- | --- | --- | --- | --- |
| ADY00129.1 | MpaB | BGC0000104 | Mycophenolic\_acid\_biosynthetic\_gene\_cluster | 41 | 95.3246753247 | 292 | 5.5e-79 |
| CDM36727.1 | Domain\_of\_unknown\_function\_DUF2236 | BGC0001360 | Mycophenolic\_acid\_biosynthetic\_gene\_cluster | 46 | 64.4155844156 | 240 | 4.2e-63 |
